# Enhancing the Photocatalytic Efficacy of g-C_3_N_4_ Through Irradiation Modification and Composite Construction with Ti_3_C_2_ for Photodynamic Therapy

**DOI:** 10.3390/molecules30030487

**Published:** 2025-01-22

**Authors:** Bin Huang, Yilun Wang, Xuguang Chen, Yue Wu, Kaidi Xu, Simeng Xie, Ziyang Qin, Xiang Liu, Huangqin Chen, Yuesheng Li

**Affiliations:** 1 Department of Stomatology, School of Stomatology and Ophthalmology, Hubei University of Science and Technology, Xianning 437100, China; huangbin914@163.com (B.H.); wangyilun04@outlook.com (Y.W.); cxg15098096179@outlook.com (X.C.); wuxiaoxiao5@outlook.com (Y.W.); kaidixu04@163.com (K.X.); 15635689920@163.com (S.X.); qzy123doc@outlook.com (Z.Q.); phosphorescent0727@gmail.com (X.L.); 2 Hubei Key Laboratory of Radiation Chemistry and Functional Materials, Non-Power Nuclear Technology Collaborative Innovation Center, Hubei University of Science and Technology, Xianning 437100, China

**Keywords:** titanium carbide, graphitic carbon nitride, electron beam irradiation, photodynamic therapy

## Abstract

Photodynamic therapy (PDT) holds considerable promise for advancing anticancer treatment, owing to its precision and minimally invasive nature. In this study, we successfully synthesized a series of titanium carbide (Ti_3_C_2_, TC)/graphitic carbon nitride (g-C_3_N_4_, CN) nanocomposite through a synergistic approach combining electron beam irradiation and 2D/2D composite formation. According to the results, 1TC/200-CN (1TC, which TC was 1, referred to the mass ratio; 200-CN, which CN was 200 kGy, referred to the irradiation metering) displayed a 94% degradation rate of methylene blue (10 mg/L) in 100 min. Furthermore, the proliferation rate of CAL-27 cells was suppressed to just 23.3% at a concentration of 320 μg/mL of 1TC/200-CN. Notably, the group treated with this concentration exhibited the largest residual scratch area, accompanied by a notable decrease in mitochondrial membrane potential. These enhanced effects were attributed to the efficient transfer of electron-hole pairs facilitated by the TC/CN composite. Our findings not only contribute to the development of efficient and stable nanocomposites for PDT applications but also provide valuable insights into the utilization of nanomaterials in the biomedical field, thereby paving the way for potential breakthroughs in cancer treatment.

## 1. Introduction

Photodynamic therapy (PDT) has garnered significant attention and research interest in the medical field as a cutting-edge therapeutic approach based on photochemical reactions. The fundamental mechanism of PDT involves the generation of reactive oxygen species (ROS) by photosensitizers under specific wavelength light irradiation. These ROS possess potent oxidizing capabilities, capable of disrupting the structure and function of target cells for therapeutic purposes [1,2]. PDT’s distinct advantages lie in its high selectivity and non-invasive nature, which have demonstrated substantial potential and broad application prospects in the treatment of dermatological, infectious, and metabolic diseases [3,4,5,6,7,8]. Illustratively, in the case of refractory chronic venous ulcers, known for their recurrent nature and slow healing process, conventional treatments often fall short. However, a carefully designed PDT regimen has led to significant reduction and accelerated healing of ulcer surfaces after 10 treatment sessions, highlighting PDT’s unique advantage in tissue repair and regeneration [9]. In the field of dermatological aesthetics, PDT has also shown remarkable efficacy in the treatment of facial flat warts. Compared to traditional methods, PDT not only significantly reduces treatment costs but also minimizes the risk of postoperative scarring and pigmentation due to its non-invasive or minimally invasive nature, offering patients a more natural and enduring aesthetic outcome [10]. In the treatment of malignant tumors, a significant health threat to humans, PDT has demonstrated considerable potential. For instance, in addressing the challenging issue of obstructive bronchial lesions in non-small cell lung cancer, PDT has effectively alleviated patients’ dyspnea and reduced the physical burden through precise targeting and minimally invasive treatment. As an integral part of a multimodal treatment strategy, PDT can act in concert with surgery, chemotherapy, and radiotherapy to collectively reduce tumor volume, mitigate surgical risks, and enhance patients’ survival and quality of life [11]. The application of PDT in this domain not only complements traditional treatment modalities but also represents a profound innovation in the conceptualization of cancer therapy [12,13,14].

Within the intricate mechanism of PDT, the selection and optimization of photosensitizers are pivotal to determining the therapeutic efficacy. Traditionally, organic photosensitizers such as porphyrins [15,16], chlorins [17,18], and phthalocyanines [19,20] have been widely utilized in PDT due to their specific optical properties and chemical structures. However, these organic photosensitizers often exhibit issues such as poor stability, limited ROS production efficiency, and inadequate tissue penetration in practical applications, which constrains their further development and utilization. Consequently, the search for and development of novel, efficient, and stable photosensitizers has become a focal point of research in the PDT field. In recent years, semiconductor nanomaterials, characterized by their unique physicochemical properties, good biocompatibility, and tunable optical performance, have emerged as a research focus in the PDT domain [21,22]. Among these, graphitic carbon nitride (g-C_3_N_4_, CN), a novel non-metallic two-dimensional semiconductor nanomaterial, has demonstrated immense potential in PDT due to its high surface area, low cost, good biocompatibility, and unique electronic structure [23,24,25]. CN is capable of generating ROS under illumination and possesses outstanding stability and processability [26,27], offering a new option for photosensitizers in PDT.

Nonetheless, the photocatalytic activity of pure CN, particularly its response efficiency in the visible light region, remains to be enhanced [28]. To surmount this limitation, researchers have employed various strategies including defect engineering, surface modification, element doping, morphology control, and composite structure formation to modify CN and enhance its photocatalytic performance [29,30,31]. Among these strategies, electron beam irradiation technology stands out as a highly controllable and flexible modification method. By adjusting parameters such as energy, dose, and irradiation time [32], it enables precise control over the surface structure of CN, thereby enhancing its light absorption capacity and ROS production efficiency [33,34]. Electron beam irradiation not only introduces defects and active sites on the CN surface but also increases its roughness and specific surface area, facilitating photocatalytic reactions. In addition to electron beam irradiation, the combination of CN with other materials is another effective approach to enhance its photocatalytic performance. Titanium carbide (Ti_3_C_2_, TC) nanosheets, as an emerging two-dimensional metal carbide material, have become an ideal composite partner for CN due to their high specific surface area, excellent near-infrared absorption capacity, high photothermal conversion efficiency, good biocompatibility, and low cytotoxicity [35,36,37,38,39]. TC nanosheets not only serve as a catalyst support to accelerate electron transfer and enhance catalytic activity but also leverage their unique physicochemical properties to confer additional advantages for PDT treatment. For instance, the antibacterial properties of TC can effectively prevent postoperative bacterial infections in cancer patients, offering an additional layer of protection for their recovery [40].

In this study, we employed electron beam irradiation technology to deeply modify CN. This process resulted in the formation of a multitude of defects and active sites on the material’s surface, while significantly enhancing its light absorption capacity. Subsequently, we utilized ultrasonic technology to combine the modified CN with TC nanosheets, forming a novel 2D/2D heterostructured nanocomposite material named 1-TC/200-CN (1TC, which TC was 1, referred to the mass ratio; 200-CN, which CN was 200 kGy, referred to the irradiation metering). We conducted an exhaustive characterization of the prepared composite material to validate its structure and investigate its photocatalytic mechanism. Through photocatalytic degradation experiments of methylene blue (MB), we evaluated the photocatalytic efficiency and stability of the 1-TC/200-CN nanocomposite material and analyzed the role of different free radicals in the photocatalytic degradation process. To further explore the potential application of the 1-TC/200-CN nanocomposite material in PDT, we conducted preliminary research using oral squamous cell carcinoma as a treatment model. Using MTT assays, Cell scratch tests, and JC-1 staining experiments, we investigated the composite material’s ability to disrupt tumor cell mitochondrial structure under illumination and inhibit cell proliferation and migration. In summary, this study successfully prepared an efficient and stable photocatalytic nanocomposite material, 1-TC/200-CN, using electron beam irradiation technology and a heterostructure construction strategy, and demonstrated its outstanding performance in the fields of photocatalytic degradation and PDT treatment. The findings of this study not only offer new research insights and methods for the PDT field but also bring new hope for the treatment of malignant tumors and other intractable diseases. We have reason to believe that with the continuous deepening of research and technological advancements, PDT will demonstrate broader application potential in the medical field, holding significant societal importance.

## 2. Results and Discussion

### 2.1. XRD and FT-IR Analysis

**Figure 1:**
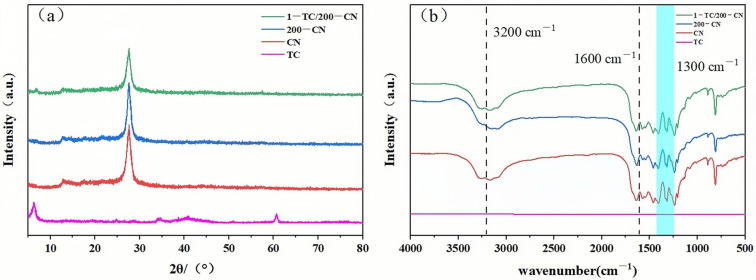
(**a**) XRD patterns of TC, CN, 200-CN, and 1-TC/200-CN; (**b**) FT-IR spectra of TC, CN, 200-CN, and 1-TC/200-CN.

The crystalline phase structures of different samples were characterized using X-ray diffraction (XRD) technique. As shown in Figure 1a, the XRD patterns of TC, CN, 200-CN, and 1-TC/200-CN are presented. TC exhibits distinct characteristic peaks at approximately 6° (002) and 62° (110). Notably, the TC diffraction peak corresponding to (002) with 2θ = 9.6° shifts to 6.4° as a result of ultrasonic treatment, indicating successful delamination of TC during the ultrasonic process [41]. CN displayed notable characteristic peaks at approximately 13° (100) and 28° (002) [42], while the characteristic peak positions of 200-CN remained unchanged, suggesting that electron beam irradiation had minimal impact on the crystalline structure of CN [34]. In this experiment, the XRD pattern of the optimally proportioned 1-TC/200-CN composite displayed corresponding characteristic peaks of both pure substances, indicating successful preparation of the composite and that the introduction of TC did not alter the structure of 200-CN.

The surface composition of the samples was analyzed using Fourier Transform Infrared (FT-IR) spectroscopy. As illustrated in Figure 1b, the FT-IR spectra of TC, CN, 200-CN, and 1-TC/200-CN are presented. It was observable that TC did not exhibit significant infrared absorption peaks. In the CN sample, the peaks at 1600 cm^−1^ and 1300 cm^−1^ were attributed to the stretching vibrations of C-N bonds and C=N double bonds, respectively, while the peak at 3200 cm^−1^ was due to the residual amino groups (-NH_2_/-NH) and water [43,44,45]. By comparing the FT-IR spectra of CN and 200-CN, it can be deduced that the radiation treatment did not alter the structure of CN. Intriguingly, the FT-IR spectrum of 1-TC/200-CN almost mirrored the peak shapes of 200-CN, with only variations in intensity, further indicating that TC did not modify the relevant structure of 200-CN during the synthesis process.

### 2.2. XPS Analysis

**Figure 2:**
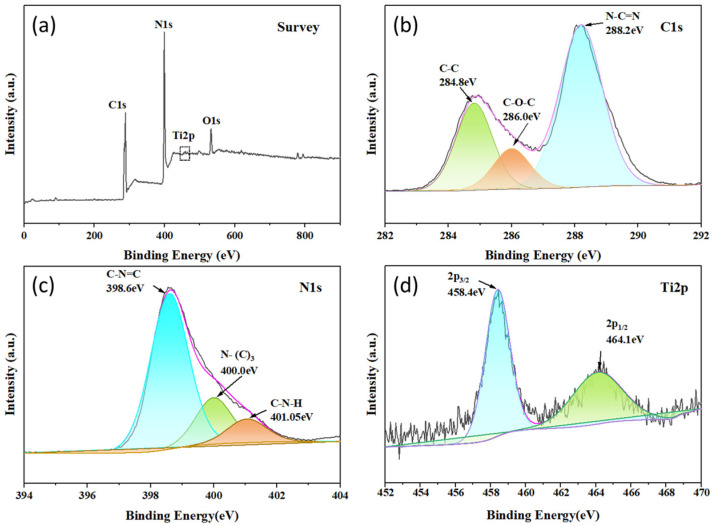
XPS spectra of 1-TC/200-CN: (**a**) XPS survey; (**b**) high-resolution XPS spectra of C 1s; (**c**) high-resolution XPS spectra of N 1s; and (**d**) high-resolution XPS spectra of Ti 2p.

The chemical element composition on the surface of the composite material was investigated using X-ray Photoelectron Spectroscopy (XPS). As shown in Figure 2a, the survey spectrum of 1-TC/200-CN revealed the presence of C, N, Ti, and O elements in the composite, where the O element may originate from oxygen in air or water. In Figure 2b, the C 1s spectrum of the composite exhibited three peaks located at 284.8 eV, 286.0 eV, and 288.2 eV, which were attributed to the C-C, C-O-C, and N-C=N bonds in CN, respectively [46,47,48]. As depicted in Figure 2c, the N 1s XPS spectrum showed three peaks at 398.6 eV, 400.0 eV, and 401.05 eV, corresponding to the C-N=C, N-(C)_3_, and C-N-H bonds, respectively [49,50]. As illustrated in Figure 2d, the Ti 2p spectrum can be divided into two peaks, with the binding energy of Ti 2p_3/2_ centered at 458.4 eV and that of Ti 2p_1/2_ at 464.1 eV [51]. The binding energies of the 1-TC/200-CN nanocomposite originated from both CN and TC, further confirming the successful preparation of the composite material.

### 2.3. FE-SEM and HR-TEM Analysis

**Figure 3:**
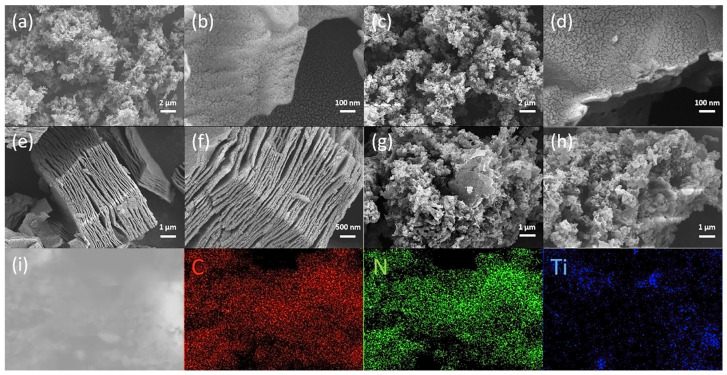
SEM images of CN (**a**,**b**), 200-CN (**c**,**d**), TC (**e**,**f**), and 1-TC/200-CN (**g**,**h**); (**i**) EDS mapping images of C, N, and Ti elements in 1-TC/200-CN.

As depicted in the accompanying figures, scanning electron microscope (SEM) images of CN, 200-CN, TC, and the 1-TC/200-CN composite were presented. Observation revealed that CN displayed a characteristic multilayer stacked structure (Figure 3a,b), whereas TC exhibited a typical accordion-like morphology (Figure 3e,f). In comparison to CN, the structural features of 200-CN remained largely unchanged (Figure 3c,d). Notably, the 1-TC/200-CN composite arose from the integration of 200-CN nanosheets with 1-TC nanosheets. Following ultrasonic treatment, 1-TC was evenly dispersed across the surface of 200-CN, leading to the formation of the 2D/2D nanoplanar composite. Furthermore, the Energy-Dispersive Spectroscopy (EDS) elemental mapping image (Figure 3i) provided additional confirmation of the uniform elemental distribution within the composite, indicating a robust interface between 200-CN and 1-TC.

**Figure 4:**
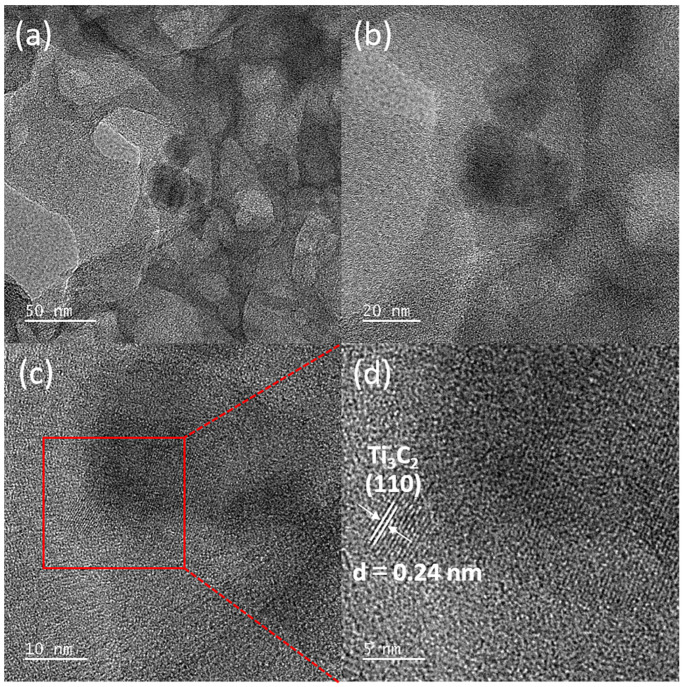
TEM (**a**,**b**) and HR-TEM (**c**,**d**) images of 1-TC/200-CN.

Additionally, Transmission Electron Microscopy (TEM) and High-Resolution Transmission Electron Microscopy (HR-TEM) analyses were conducted on the 1-TC/200-CN composite, further elucidating the typical 2D/2D stacked nanosheet morphology of the synthesized composite. As shown in TEM images (Figure 4a,b), the composite consists of two layered nanomaterials, with the brighter regions corresponding to 200-CN and the darker regions to 1-TC, suggesting a close interface between 200-CN and 1-TC [52]. HR-TEM images (Figure 4c,d) provided more detailed insights, revealing a lattice spacing of 0.24 nm attributable to the (110) plane of 1-TC, while the amorphous regions correspond to 200-CN. This reaffirmed the successful preparation of the 2D/2D 1-TC/200-CN nanosheet composite [41] and was consistent with the results obtained from XRD and SEM.

### 2.4. Analysis of Photocatalytic Activity

**Figure 5:**
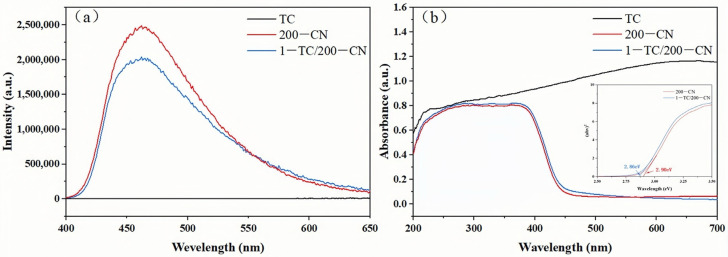
(**a**) PL emission spectra of TC, 200-CN, and 1-TC/200-CN; (**b**) DRS spectra of TC, 200-CN, and 1-TC/200-CN.

The separation efficiency of photogenerated carriers was investigated using Photoluminescence (PL) spectroscopy. With an excitation wavelength of 320 nm, the PL intensity of the 1-TC/200-CN composite was studied. As shown in Figure 5a, TC exhibited no significant emission peaks due to its inherent physicochemical properties [53]. Compared to 200-CN, the PL intensity of 1-TC/200-CN was significantly reduced, indicating that the composite effectively promotes the separation of electrons and holes, thereby enhancing photocatalytic performance [54]. The UV-Visible Diffuse Reflectance Spectra (DRS) of TC, 200-CN, and 1-TC/200-CN were presented in Figure 5b. Compared to the absorption edge of 200-CN at 450 nm, 1-TC/200-CN displayed a notable band edge shift, attributed to the broad-spectrum absorption capability of TC, thereby enhancing the photocatalytic performance of the composite [55]. Calculations using the Kubelka–Munk function revealed a band gap energy of 2.90 eV for 200-CN. Upon combination with TC, the band gap energy shifts from 2.90 eV to 2.86 eV. This narrowing of the forbidden band gap suggested that the composite can absorb more visible light, thereby exhibiting superior photocatalytic capability.

### 2.5. Photocatalytic Degradation of Organic Dyes

**Figure 6:**
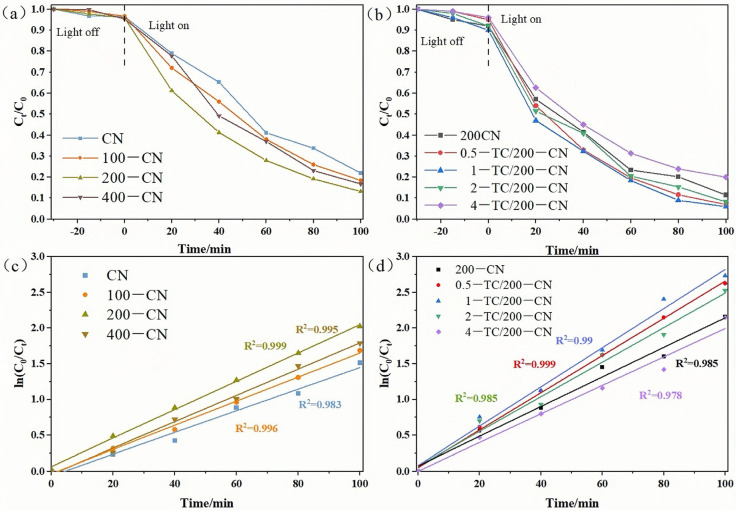
(**a**,**b**) Photocatalytic performance of various photocatalysts for the degradation of MB under visible light irradiation; (**c**,**d**) Pseudo-first-order kinetic curves for the degradation of MB under visible light irradiation.

MB dye was a common cationic dye known for its environmental persistence, toxicity, carcinogenic properties, and mutagenicity. At doses greater than 5 mg/kg, MB’s monoamine oxidase inhibitory characteristics posed a threat not only to aquatic organisms within aquatic ecosystems but also had the potential to induce fatal serotonin toxicity in humans. Therefore, the removal of MB dye from wastewater was highly necessary. CN-based materials were commonly modified for applications in dye degradation [56,57]. In this study, the influence of modification strategies such as irradiation technology and the formation of complexes with TC on the performance of CN in degrading the organic dye MB was also investigated. In the degradation experiment, an adsorption–desorption equilibrium of MB dye was achieved through a 30 min dark reaction, with negligible changes in MB solution concentration before and after the reaction. Figure 6a illustrated that electron beam radiation modification significantly enhanced the photocatalytic performance of CN, with 200-CN exhibiting the optimal photocatalytic activity. Figure 6b showed the influence of composites formed by combining 200-CN with TC at different mass ratios on photocatalytic performance. When the doping mass ratio of TC is 0.5, 1, and 2, the photocatalytic activity significantly improved, with 1-TC/200-CN displaying the highest photocatalytic activity, achieving a degradation rate of 94% within 100 min. The possible reason for this phenomenon was that as the TC content increased, the close contact between the lamellar planes enhanced charge separation, reducing electron-hole pair recombination and thereby enhancing photocatalytic performance. Notably, when the mass ratio is 4, the photocatalytic activity of the composite was lower than that of 200-CN. This may be due to excessive TC shielding the active sites of 200-CN, creating a physical barrier during the catalytic process that reduced the surface area involved in the catalytic reaction, thereby decreasing the photocatalytic efficiency.

The kinetics of MB photodegradation over the catalysts can be described using a first-order equation:

(1) $$C_{t}=C_{0}\times e^{-kt}$$

where *C_0_* represented the initial dye concentration, *C_t_* represented the dye concentration at time *t*, and *k* was the first-order reaction rate constant. Figure 6c and 6d presented the kinetic curves for the degradation of MB by different catalysts, with detailed data provided in Table 1 and Table 2, respectively. Comprehensive data analysis revealed that 200-CN exhibited the highest degradation rate in Figure 6c, with a reaction rate constant of 0.01982 min^−1^. In Figure 6d, 1-TC/200-CN demonstrated the highest degradation rate, with a reaction rate constant of 0.02740 min^−1^, which was 1.8 times and 1.4 times higher than that of CN (0.01511 min^−1^) and 200-CN (0.01982 min^−1^), respectively. This indicated that the composite material significantly enhanced the photocatalytic efficiency of CN. However, some scholars believe that the degradation of organics through photocatalysis may generate secondary pollutants, which restricts their comprehensive industrial application. In contrast, adsorption technology had been found to have more advantages compared to other methods [58].

**Figure 7:**
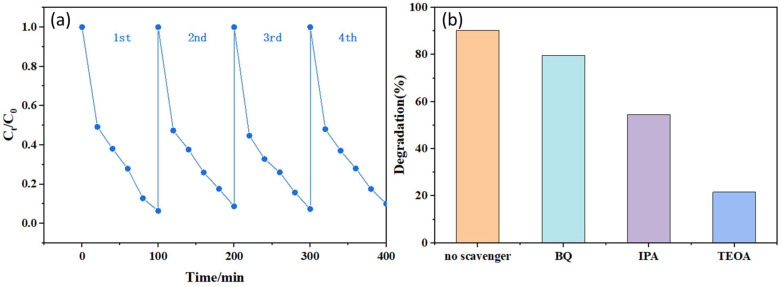
(**a**) Cyclic stability experiment of 1-TC/200-CN for the degradation of MB; (**b**) Effect of different scavengers on the degradation rate of MB by 1-TC/200-CN.

To further investigate the cyclic stability of the 1-TC/200-CN composite and the primary active species involved in its photocatalytic degradation process, we conducted cyclic experiments for photocatalytic degradation of MB dye and radical trapping experiments. In the cyclic experiments, the preliminary work is consistent with the photocatalytic degradation experiment. After each experiment, the samples are repeatedly washed with ethanol and water, centrifuged, and then dried to collect the samples. We evaluated the photocatalytic degradation rate of the composite after four consecutive cycles. As shown in Figure 7a, the degradation rate of MB remained at 90% after four cycles, indicating the excellent chemical stability of 1-TC/200-CN. In the radical trapping experiments, triethanolamine (TEOA), isopropanol (IPA), and β-benzoquinone (BQ) were used as scavengers for holes (h+), hydroxyl radicals (·OH), and superoxide radicals (·O_2_^−^), respectively, with a scavenger concentration of 0.5 mmol/L. As depicted in Figure 7b, the degradation rate of MB decreased after the addition of all three different scavengers, suggesting that h+, ·OH, and ·O_2_^−^ all play significant roles in the degradation process. Compared to the other two scavengers, the addition of triethanolamine (TEOA) resulted in a substantial reduction in MB degradation efficiency, indicating that h+ play a crucial role in the degradation process.

**Figure 8:**
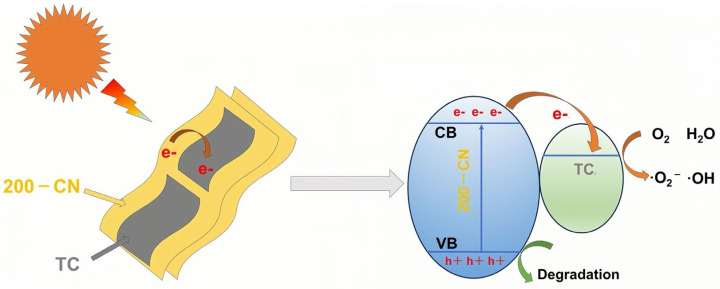
Schematic diagram of photocatalytic mechanism of 1-TC/200-CN.

Under solar irradiation, the photocatalytic mechanism of 1-TC/200-CN is illustrated as shown in Figure 8. TC and 200-CN are closely attached together in a lamellar form. Under light exposure, 200-CN is excited to produce a large number of electrons and holes. Due to the close contact between the interfaces and the excellent physicochemical properties of TC, the photogenerated electrons of 200-CN quickly migrate to the surface of TC. This greatly promotes the separation of photoexcited carriers in the 1-TC/200-CN composite material. Subsequently, the electrons aggregated on the surface of TC participate in the photocatalytic degradation reaction, significantly enhancing the photocatalytic degradation performance of the composite material for organic dyes.

### 2.6. Effects on CAL-27 Cell Proliferation

**Figure 9:**
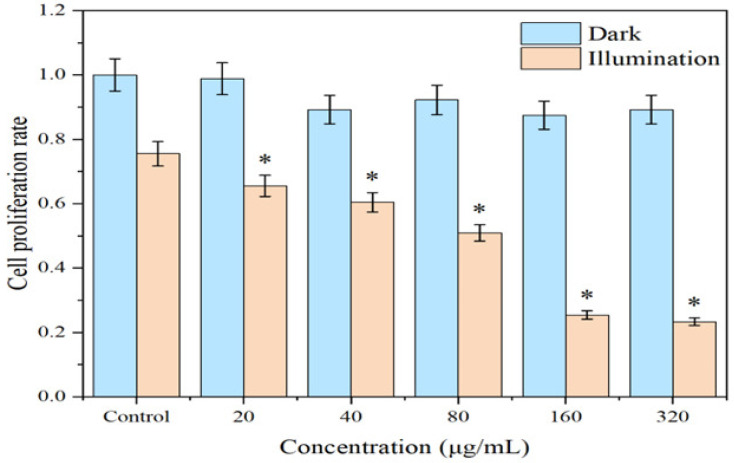
Effect of 1-TC/200-CN composite on the proliferation of CAL-27 cells. CAL-27 cells were co-cultured with varying concentrations of 1-TC/200-CN composite for 24 h and assayed using the MTT method. *: *p* < 0.05 compared to the light-exposed control group.

The MTT assay was utilized to evaluate the impact of various concentrations of the 1-TC/200-CN composite on the proliferation of CAL-27 cells under both illuminated and dark conditions. As depicted in Figure 9, under dark conditions, the proliferation activity of CAL-27 cells did not decrease significantly with increasing concentrations of the material. However, under illuminated conditions, a notable decline in CAL-27 cell proliferation activity was observed across all concentrations. Specifically, at a material concentration of 320 μg/mL, the cell proliferation rate was the lowest, reaching only 23.3%. These results indicated that under illuminated conditions, the 1-TC/200-CN composite exerted a significant inhibitory effect on CAL-27 cell proliferation, and this effect exhibits a concentration-dependent trend.

### 2.7. Impact on the Lateral Migration of CAL-27 Cells

**Figure 10:**
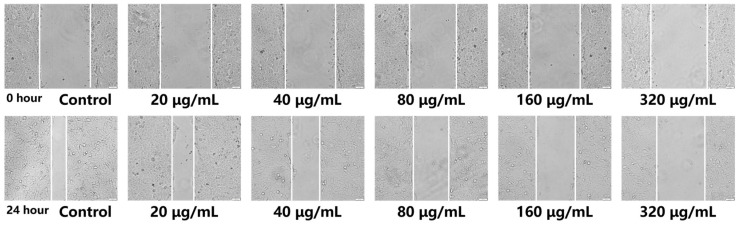
Effect of different concentrations of 1-TC/200-CN composite on cell migration. The scratch area was observed under a microscope (×10 magnification). 0 h: Before the addition of the composite; 24 h: 24 h after the addition of the composite.

The cell scratch assay was a commonly used method for detecting the migration ability of cells. The fundamental principle of this technique involved creating an artificial “wound” on a culture dish. By capturing images at regular intervals and utilizing software such as Image J (version 18.0) to calculate the wound area, the ability of cells to migrate and proliferate into the wounded area over time can be observed. This, in turn, evaluated cellular activity and wound healing capacity [59]. In this study, the cell scratch assay was conducted to evaluate the effect of various concentrations of 1-TC/200-CN composites under photodynamic action on the migration capacity of CAL-27 cells. As illustrated in Figure 10, at the 0 h time point, the scratch area of all groups was approximately 13,390 ± 690.5 pixel^2^, with no significant differences. After 24 h, a reduction in scratch area was noted in all groups. Among them, the group with a concentration of 320 μg/mL exhibited the largest remaining scratch area (11455 ± 572.75 pixel^2^), while the control group had the smallest (3915 ± 195.75 pixel^2^). These findings indicated that after 24 h of incubation, the 1-TC/200-CN composites significantly inhibited the lateral migration of CAL-27 cells, demonstrating a concentration-dependent effect.

### 2.8. Impact on Mitochondrial Membrane Potential of CAL-27 Cells

**Figure 11:**
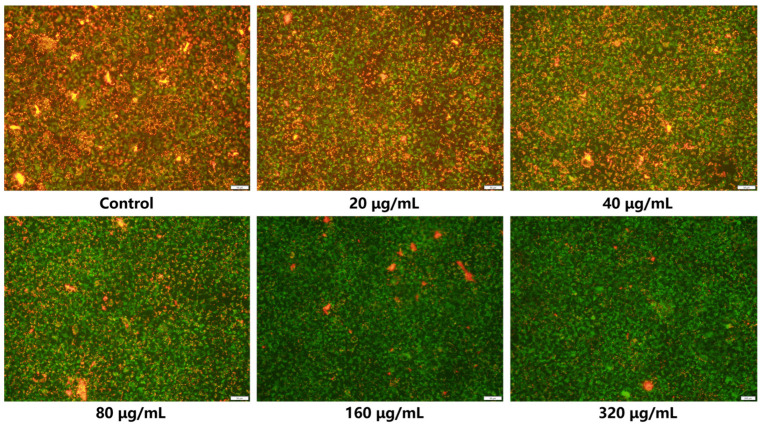
Effect of different concentrations of 1-TC/200-CN composite on mitochondrial membrane potential in CAL-27 cells. Cells were stained with JC-1 and observed under a microscope (×4 magnification).

JC-1 staining was an experimental technique used to detect changes in mitochondrial membrane potential within cells. It formed aggregates of different colors depending on the membrane potential, reflecting the level of mitochondrial membrane potential through its distribution within mitochondria [60]. Under normal conditions, healthy mitochondria exhibited red fluorescence (JC-1 aggregates). When mitochondria are damaged, the mitochondrial membrane potential decreased, resulting in the appearance of green fluorescence (JC-1 monomers) within the mitochondria [61]. Therefore, in this study, the JC-1 staining method was employed to assess the effect of various concentrations of 1-TC/200-CN composites on the mitochondrial membrane potential of CAL-27 cells under photodynamic action. As shown in Figure 11, the control group exhibited strong red fluorescence and weak green fluorescence, whereas the 320 μg/mL group displayed an opposite pattern. Furthermore, as the concentration of the composites increased, the green fluorescence in the mitochondria became increasingly intense, while the red fluorescence gradually weakened, demonstrating a clear concentration-dependent trend. These results indicated that the 1-TC/200-CN composite material effectively induced apoptosis in CAL-27 cells under photodynamic action, with the mechanism closely linked to a significant decrease in mitochondrial membrane potential within the cells. Specifically, the composite material may exert its effect by interfering with or inhibiting the normal function of the main mitochondrial electron transport chain (ETC) complexes [62]. This interference may manifest as impacting the electron transfer efficiency between complexes or altering their redox states, thereby achieving precise regulation of the mitochondrial membrane potential. Ultimately, this series of actions lead to the apoptotic process in CAL-27 cells.

## 3. Materials and Methods

### 3.1. Preparation of TC/CN Composites

CN was synthesized via a high-temperature calcination method. Initially, 10 g of urea (Sinopharm Chemical Reagent Co., Ltd., Shang Hai Shi, China) were dissolved in 20 milliliters of deionized water and subjected to ultrasonic treatment (the frequency is 40 KHz and the power is 360 W) in an ice-water bath for two hours to obtain a uniformly dispersed urea suspension. Subsequently, the suspension was vacuum-dried at 60 °C for 24 h to procure the precursor. The precursor was then placed in a tubular furnace and calcined at 550 °C under a nitrogen atmosphere with a heating rate of 10 °C/min for 2 h to yield the final product.

To prepare the modified CN, 0.12 g of CN were mixed with 12 mL of isopropanol, 120 mL of deionized water, and 240 mL of ammonia in a beaker. This mixture was then sonicated in an ice-water bath for 30 min to ensure uniform dispersion. The mixture was transferred into a polyethylene bag and sealed under vacuum to remove any air bubbles. The sample was subsequently irradiated using a 1 MeV electron accelerator (1 MeV; Wasik Associates, Dracut, MA, USA) at a dose rate of 20 kGy per pass. After undergoing three cycles of washing with ethanol and deionized water, the sample was vacuum-dried at 60 °C and then grinded for further use. The samples subjected to different irradiation doses were denoted as X-CN, where X represents the irradiation dose, with X values of 100, 200, and 400 kGy.

Single-layer TC was obtained using an ultrasonic method. Multilayer clay-like TC (Foshan Xinxi Technology Co., Ltd., Foshan, China) was placed in 10 mL of deionized water and subjected to ultrasonic treatment in an ice-water bath for 15 min. The obtained samples were denoted as T-TC, where T represents the mass ratio of TC to CN, specifically with T values of 0.5, 1, 2, and 4. Pre-experimental results indicated that CN exhibited optimal photocatalytic efficiency when irradiated at a dose of 200 kGy. Therefore, T-TC/200-CN composites were prepared using an ultrasonic method. Specifically, 0.1 g of 200-CN was dispersed in 10 milliliters of deionized water and sonicated in an ice-water bath for 2 h. This dispersion was then mixed with the suspension of T-TC and further sonicated for an additional 4 h. Following centrifugation, the resultant precipitate was vacuum-dried at 60 °C for 24 h to obtain the final products, which were labeled as T-TC/200-CN. Preliminary experiments have indicated that TC/200-CN exhibits optimal photocatalytic efficiency when the mass ratio is 1. Consequently, the 1TC-200CN composite was utilized for all subsequent experiments.

### 3.2. Material Characterization

The phase structure of the samples was analyzed using XRD (the voltage is 40 kilovolts, the scanning range is from 5.000 to 80.000, the scanning mode is continuous scanning, and the scanning speed is 5.000 (degrees per minute)). FT-IR with potassium bromide particles was employed to investigate the chemical composition of the samples. The morphology and elemental analysis of the samples were studied using SEM, (Zeiss Gemini Sigma 300, Wuhan, Hubei, China) and TEM (JEM-F200, Wuhan, Hubei, China). XPS of the samples was conducted on an XPS spectrometer (Thermo Fisher Scientific Nexsa, Wuhan, Hubei, China). PL spectra of the samples were recorded using a fluorospectrometer (Edinburgh FLS-1000, Wuhan, Hubei, China) with an excitation wavelength of 320 nm. UV-Vis diffuse reflectance spectra of the samples were obtained using a UV-Vis spectrophotometer (Shimadzu 3600-plus, Wuhan, Hubei, China) with a scanning range of 200–800 nm.

### 3.3. Photocatalytic Degradation Experiments

MB was utilized as a model compound to evaluate the photocatalytic activity of the samples. Thirty milligrams of the sample were dispersed in fifty milliliters of MB solution with a concentration of 10 mg/L. Under stirring conditions, the sample was allowed to adsorb in the dark for 30 min to ensure an adsorption–desorption equilibrium between the sample and the MB dye. The photocatalytic experiments were conducted under a deuterium lamp (The distance between light source and reaction vessel was 15 cm, light intensity or flux was 500 W, wavelength > 420 nm). During the experiment, 3 mL of solution was withdrawn from each suspension every 20 min. The photocatalyst was separated using a high-speed centrifuge (8000 rpm, 3 min), and the MB dye concentration at different time points was measured using a UV-Vis spectrophotometer at λ_max_ = 664 nm. The degradation rate was calculated using the following formula:

(2) $$Degradation\;Rate\left(\%\right)=\frac{C_{0}-C_{t}}{C_{0}}\times 100\%=\frac{A_{0}-A_{t}}{A_{0}}\times 100\%$$

Note: *C_0_* and *C_t_* represent the dye concentrations at the initial time and time *t*, respectively, while *A_0_* and *A_t_* represent the dye absorbances at the initial time and time *t,* respectively.

### 3.4. MTT Assay

CAL-27 cells were seeded in 96-well plates and incubated for 12 h. After cell adhesion, 100 μL of 1-TC/200-CN solutions with various concentrations (20, 40, 80, 160, 320, 640 μg/mL) were added to each well, except for the control group. The cells were then irradiated using a light source with an intensity of 0.45 W/cm^2^ and a wavelength of 670 nm (5 min of irradiation followed by a 2 min break), for a total irradiation time of 10 min. Following irradiation, the cells were further incubated for 24 h. Each well was then treated with 10 μL of MTT solution (5 mg/mL) and incubated for an additional 4 h. The supernatant was removed, and 150 μL of DMSO was added. The absorbance of each well was measured at a wavelength of 490 nm using a microplate reader. By comparing with the control group, the effect of 1-TC/200-CN on CAL-27 cell proliferation could be effectively demonstrated.

### 3.5. Cell Scratch Assay

Using a marker pen, lines were drawn at intervals of 0.5–1 cm on the reverse side of a 6-well plate. CAL-27 cells were seeded into the 6-well plate and incubated overnight. Multiple scratches were made on the cell monolayer using a scratching tool or scraper along the marker pen traces. The scratched areas were then washed with PBS solution to remove cellular debris. Immediately after scratching, photographs were taken using microscope and recorded as 0 h. Subsequently, except for the control group, 1 mL of 1-TC/200-CN solution at different concentrations (20, 40, 80, 160, 320 μg/mL) was added to each well, ensuring that the scratches were covered. Irradiation was performed using a light source with an intensity of 0.45 W/cm^2^ and a wavelength of 670 nm (with a 2 min break after every 5 min of irradiation), for a total irradiation time of 10 min, followed by continued incubation for 24 h. After the incubation period, the same areas were observed again, and the width of the wounds was measured.

### 3.6. JC-1 Staining

CAL-27 cells were seeded into a 6-well plate. Once the cells adhered, 1 mL of 1-TC/200-CN solution at different concentrations (20, 40, 80, 160, 320 μg/mL) was added to each well, except for the control group. The cells were then irradiated using a light source with an intensity of 0.45 W/cm^2^ and a wavelength of 670 nm (with a 2 min break after every 5 min of irradiation), for a total irradiation time of 10 min, followed by continued incubation for 24 h. Subsequently, the cells were stained with JC-1 staining solution and incubated for an additional 20 min. Afterward, the cells were rinsed twice with JC-1 staining buffer. The cells were observed and photographed under an inverted fluorescence microscope. In normal mitochondria, JC-1 aggregates within the mitochondria to form polymers, emitting red fluorescence. However, in damaged mitochondria with decreased membrane potential, JC-1 exists as monomers within the cells, displaying green fluorescence. The mitochondrial membrane potential was assessed by examining the fluorescence pattern and intensity.

## 4. Conclusions

In this study, we successfully prepared a 2D/2D heterojunction 1-TC/200-CN nanocomposite with optimal photocatalytic performance by utilizing electron beam irradiation technology and forming a heterojunction with Ti3C2 material. Photocatalytic degradation experiments demonstrated that the degradation rate of this composite was 1.8 times higher than that of the CN material. Detailed characterization illuminated that this remarkable enhancement was primarily due to the synergistic effect of two modification strategies. These strategies not only augmented the active sites and specific surface area of the material but also expedited the transfer of electron-hole pairs, thereby significantly bolstering the photocatalytic prowess of the composite.

Subsequently, using CAL-27 cells as a model, we validated that under light illumination, the 1-TC/200-CN nanocomposite could markedly suppress cellular proliferation and migration by disrupting mitochondrial structure. This underscores the immense potential of our composite in PDT for anticancer treatment. More significantly, it not only furnishes an efficient and stable nanocomposite for PDT, promising to elevate treatment efficacy and mitigate treatment costs in practical clinical settings, but also presents novel research paradigms and methodologies for nanomaterials within the biomedical realm.

## Figures and Tables

**Table 1:** Degradation rates of MB by CN modified with different irradiation doses.

	k (min^−1^)	R^2^	Degradation Rate (%)
CN	0.01511	0.983	78.0
100-CN	0.01683	0.996	81.5
200-CN	0.01836	0.999	86.8
400-CN	0.01982	0.995	83.2

**Table 2:** Degradation rates of MB by composite materials with various TC mass ratios.

	k (min^−1^)	R^2^	Degradation Rate (%)
200-CN	0.02071	0.985	88.5%
0.5-TC/200-CN	0.02505	0.999	93.0%
1-TC/200-CN	0.02740	0.990	94.0%
2-TC/200-CN	0.02419	0.985	91.7%
4-TC/200-CN	0.019992	0.978	80.0%

## Data Availability

Data are contained within the article.
